# Macrophages Support Splenic Erythropoiesis in 4T1 Tumor-Bearing Mice

**DOI:** 10.1371/journal.pone.0121921

**Published:** 2015-03-30

**Authors:** Min Liu, Xing Jin, Xigan He, Ling Pan, Xiumei Zhang, Yunxue Zhao

**Affiliations:** 1 Department of Pharmacology, School of Medicine, Shandong University, Jinan, 250012, China; 2 Qilu Hospital, Shandong University, Jinan, 250012, China; Rutgers - New Jersey Medical School, UNITED STATES

## Abstract

Anemia is a common complication of cancer; a role of spleen in tumor-stress erythropoiesis has been suggested. However, the molecular mechanisms involved in the splenic erythropoiesis following tumor maintenance remain poorly understood. Here we show that tumor development blocks medullar erythropoiesis by granulocyte colony-stimulating factor (G-CSF) and then causes anemia in murine 4T1 breast tumor-bearing mice. Meanwhile, tumor-stress promotes splenic erythropoiesis. Splenectomy worsened tumor-induced anemia, and reduced tumor volume and tumor weight, indicating the essential role of spleen in tumor-stress erythropoiesis and tumor growth. Tumor progression of these mice led to increased amounts of bone morphogenetic protein 4 (BMP4) in spleen. The in vivo role of macrophages in splenic erythropoiesis under tumor-stress conditions was investigated. Macrophage depletion by injecting liposomal clodronate decreased the expression of BMP4, inhibited splenic erythropoiesis, aggravated the tumor-induced anemia and suppressed tumor growth. Our results provide insight that macrophages and BMP4 are positive regulators of splenic erythropoiesis in tumor pathological situations. These findings reveal that during the tumor-stress period, the microenvironment of the spleen is undergoing changes, which contributes to adopt a stress erythropoietic fate and supports the expansion and differentiation of stress erythroid progenitors, thereby replenishing red blood cells and promoting tumor growth.

## Introduction

Erythropoiesis is a process through which the hematopoietic stem cells (HSCs) develop into red blood cells (RBCs) [1]. Under physiologic conditions, erythropoiesis is regulated by a complex feedback mechanism to maintain the mass of circulating RBCs at an optimum level [2, 3]. Anemia of inflammation (AI), one of the most common forms of anemia seen clinically, develops during chronic inflammatory conditions, such as chronic infections, malignancies, and chronic kidney disease [4]. Cancer, one of the leading causes of death worldwide, continues to be a challenging and complex disease [5]. Patients with cancer frequently suffer from a multifactorial anemia, which negatively influences normal mental and physical functions with debilitating symptoms, such as fatigue, headache and depression. The etiology and treatment for cancer-related anemia remain controversial, and the complexity of the interactions between tumors and erythropoiesis system has been examined and discussed [6, 7]. The tumor progression is often associated with inflammatory response [8], which may lead to anemia [9, 10]. Tumor cells have been reported to express granulocyte colony-stimulating factor (G-CSF) [11, 12] that can mobilize HSCs from bone marrow and suppress medullar erythropoiesis [13, 14].

At steady state, medullar erythropoiesis is primarily homeostatic, whereas stress erythropoiesis predominates in the spleen after several disorders, such as acute anemia [15], AI [16], polycythemia vera and β-thalassemia [17]. The spleen contains a unique microenvironment that can support stress-erythropoiesis; splenic erythropoiesis requires bone morphogenetic protein 4 (BMP4) to stimulate expansion of stress-erythroid progenitors in the spleen and promote their differentiation into burst-forming units-erythroid (BFU-E) that respond to high levels of Epo and differentiate rapidly into erythroblasts [15, 16, 18]. Anemia and splenic erythropoiesis associated with splenomegaly have been shown in various murine tumor models [11, 19]. However, the molecular processes responsible for splenic erythropoiesis in tumor-bearing mice have remained elusive.

Definitive erythropoiesis is performed at erythroblastic island, which is composed of a central macrophage surrounded by erythroblasts at different stages of maturation [20]. Erythroblastic islands have been observed in bone marrow, splenic red pulp and fetal liver. Macrophages within these structures carry out essential physiological functions in promoting erythroblast survival, proliferation and terminal differentiation, as well as enucleation of late stage erythroblasts. These effects depend on erythroblast-macrophage interaction, which include the function of adhesion molecules and factors secreted within the erythroblastic island [21, 22]. Macrophages also play a crucial role in stress erythropoiesis that develops in the spleen. Under conditions of stress erythropoiesis, splenic macrophages synthesize BMP4 in response to Epo, which induces proliferation of stress BFU-E [16]; chemical ablation of splenic macrophage decreases the expression of BMP4 and impairs splenic erythropoiesis [17, 23]. Nonetheless, whether and how macrophages contribute to splenic erythropoiesis in tumor-bearing mice remain to be elucidated. To get a better insight into the link between cancer, anemia and erythropoiesis, we examined the role of spleen in 4T1 breast tumor-bearing mice. Notably, tumor-bearing mice with splenectomy display severe anemia, which indicates a decisive contribution of spleen in tumor stress erythropoiesis. Moreover, we explored the role of macrophages to splenic erythropoiesis and the effect of pathological splenic erythropoiesis on tumor growth, and our findings highlight the significance of splenic erythropoiesis in tumor progression.

## Materials and Methods

### Murine 4T1 breast tumor model

Mouse breast tumor cells (4T1) were purchased from Shanghai Cell Bank, Type Culture Collection Committee, Chinese Academy of Sciences (cat number: TCM32). The cells were cultured in RPMI-1640 medium supplemented with 10% heat inactivated FBS, 2mM glutamine, 100U/ml penicillin, 100μg/ml streptomycin and maintained at 37°C in a humidified atmosphere of 5% CO_2_. The mice were purchased from laboratory animal center of Shandong University. Female BALB/c mice, 6–8 weeks, were anesthetized with sodium pentobarbital. 4T1 cells (5×10^5^) in 100μL RPMI-1640 medium were implanted into the right fourth mammary gland. Tumor volume was calculated using the following formula: [L×W^2^]/2 (L = length of tumor; W = width of tumor). On day 28 of 4T1 cells injection, mice were sacrificed by cervical dislocation, and the tumors were collected and weighed. All the protocols of experiments were approved by the Institutional Care and Use Committee of Shandong University (Permit Number: 201402079) and performed according to the Guide for the Care and Use of Laboratory Animals published by the US National Institutes of Health.

### Hematologic analysis

Mice were anesthetized with sodium pentobarbital. Blood was collected on ethylenediaminetetraacetic acid (EDTA) by cardiac puncture. Red blood cell (RBC) counts, reticulocytes, hemoglobin (Hb) concentrations and hematocrit were measured on the SYSMEX XT-2000iV automated blood cell analyzer.

### Surgical removal of the spleen

Skin covering the spleen was shaved and cleaned with 70% ethyl alcohol. A splenectomy was performed under sodium pentobarbital anesthesia by making an incision in the dorsal left flank. After the splenic vessels were ligated with silk, the spleen was removed. The incision was thereafter sutured. In the sham-operated group, mice were anesthetized and back operation performed but the spleen was not removed. The operated mice were allowed to recover for at least 2 weeks before the onset of experiments.

### Measurement of RBC lifespan

RBC lifespan was determined by labeling RBCs with sulfo-NHS-biotin reagent as described with minor modifications [17]. A single dose of 1mg of sulfo-NHS-biotin was injected into mice intravenously at day 7 after tumor cells transplantation. On days 8, 15, 22 and 29 of tumor cells transplantation, a drop of blood was collected from the tail, and the blood cells were washed three times in phosphate-buffered saline supplemented with 0.1% glucose. The erythrocytes (adjusted to 3 million cells) were incubated with fluorescein-conjugated streptavidin at 25°C for 1 hour, and analyzed by flow cytometry. The percentage of biotinylated cells was calculated as a ratio of positive cells to all RBCs.

### Flow cytometry analysis of mouse erythroid cells

Flow cytometry analysis for erythroid cells was done as described with minor modifications [16, 17]. Splenocytes were isolated by mechanical dissociation of the spleen, bone marrow cells were flushed out of femurs, and cells were resuspended in Iscove’s medium. After preincubation with 1 μg/mL rat IgG for 10 min at 4°C, cells were immunostained for 30 minutes at 4°C with FITC-conjugated anti-Ter119 and phycoerythrin-conjugated anti-CD71 antibodies. After staining, cells were washed with PBS, and the samples were analyzed on a flow cytometer.

### BMP4 quantitative reverse-transcribed polymerase chain reaction

Total RNA was isolated from splenocytes, homogenized in TRIzol, and reverse transcribed into cDNA. qPCR was performed with SYBR GREEN on an CFX96 Real-Time System. The PCR protocol consisted of one cycle at 95°C (3 min) followed by 40 cycles of 95°C (15 s) and 55°C (1 min). Expression of the mouse ribosomal S14 mRNA was used as a standard. The average threshold cycle number (CRtR) for each tested mRNA was used to quantify the relative expression of each gene: 2^Ct (S14) −Ct^ (gene). The primers used were BMP4 forward, ATTCCTGGTAACCGAATGCTG, BMP4 reverse, CCGGTCTCAGGTATCAAACTAGC; S14 forward, CAGGACCAAGACCCCTGGA, S14 reverse, ATCTTCATCCCAGAGCGAGC [16, 23].

### Immunohistochemistry and immunofluorescence

Spleens were harvested, cut into two halves along the longitudinal axis, and fixed in 10% buffered formalin and embedded in paraffin. Paraffin-embedded spleens were sectioned for immunohistochemistry. After rehydration, longitudinal sections (4 μm) were incubated in 0.3% H_2_O2 in water for 10 min, then with 10 mM sodium citrate/0.05% Tween-20 (pH 6.0) in a microwave oven for antigen retrieval. Sections were blocked with 5% PBS-bovine serum albumin for 30 min at room tempreture, followed by labeling with BMP4 antibody overnight at 4°C. Remaining steps were performed using GTVision kits. Slides were counterstained with hematoxylin. Pictures were obtained using Zeiss microscope. Immunofluorescence was performed for analysis of BMP4 and F4/80. Sections of spleen were permeabilized with 0.1% Triton X-100, washed with PBS and blocked with 5% PBS-bovine serum albumin. Then sections were stained with rabbit antibody to mouse BMP4 overnight at 4°C. After washing with PBS, slides were stained with Alexa fluor 488 conjugate anti-rabbit secondary antibody and phycoerythrin-conjugated antibody to F4/80, along with DAPI for 1 hr at RT. Images were acquired using Zeiss confocal microscope LSM780 and processed with Zeiss LSM Image browser software.[16, 23]

### Depletion of macrophages with liposomal clodronate in vivo

Cancer cells (4T1) were implanted into the right fourth mammary gland of female BALB/c mice (day 0). After tumor cells injection, mice were intravenous (i.v.) injected with liposomal clodronate 100 μL (7 mg/ml) on days 7 and 18. The control mice were injected with control liposome.

### BFU-E and CFU-E colony assay

Splenocytes and bone marrow cells were isolated from control and tumor-bearing mice. Then, 5×10^4^ nucleated bone marrow and 5×10^5^ nucleated splenocytes were plated in methylcellulose media, according to the instructions of manufacturer. BFU-E and CFU-E colonies were counted under a microscope on days 14 and 8 of culture.

### Measurement of serum cytokine concentration

Mice were anesthetized with sodium pentobarbital. Blood was collected by cardiac puncture and allowed to clot at room temperature for 1.5 hr. Each sample was centrifuged to pellet the RBCs, and serum was collected and stored at -80°C. Determination of serum concentrations of Epo, IL-6, INF-γ and TNF-α were carried out using commercially available enzyme-linked immunosorbent assay kits obtained from BD or R&D Systems, according to the manufacture’s instructions. G-CSF concentrations were measured by flow cytometry using the mouse G-CSF Flex-Set bead array.

### Serum iron parameters

Serum iron and unsaturated iron binding capacity were measured using an Iron/TIBC reagent set, and transferrin saturation was calculated according to the manufacture’s instructions.

### Granulocyte colony-stimulating factor (G-CSF) treatment

Mice were injected twice daily subcutaneously with 125 μg/kg of recombinant human G-CSF or saline for up to 21 consecutive days.

### Statistical analysis

Results are expressed as mean ± SEM. Analyses of different treatment groups were conducted using analysis of variance (ANOVA) and Student t test with the Prism software (GraphPad Software, Inc.). P values< 0.05 were considered statistically significant, and p< 0.01 was regarded as highly significant.

## Results

### Tumor-bearing mice display anemia and high serum levels of G-CSF

**Fig 1:**
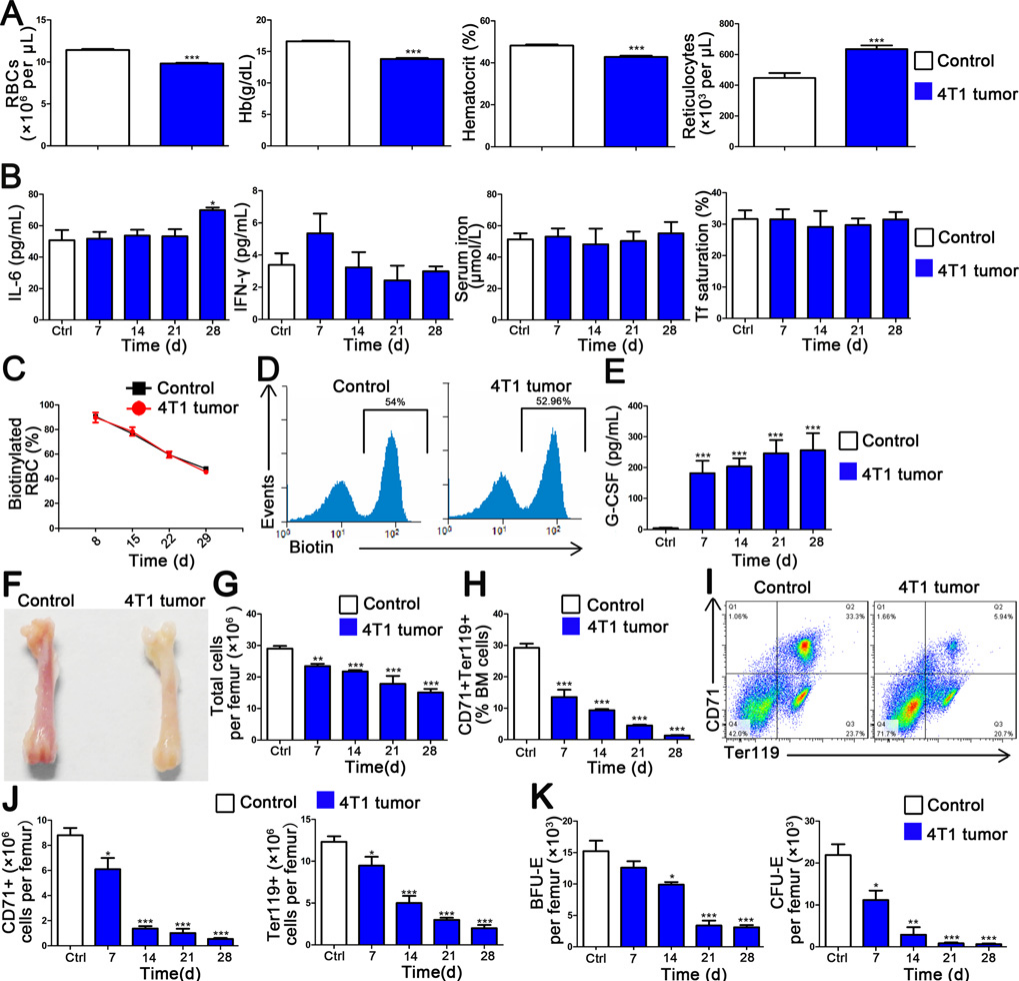
Tumor-bearing mice display anemia and bone marrow erythropoiesis repression. (A) Hematological parameters in 4T1 tumor-bearing mice. BALB/c mice were inoculated 4T1 cells and tumors were grown for 28 days. Blood was collected from the mice, and RBC counts, reticulocytes, hemoglobin (Hb) concentrations and hematocrit were measured on the automated blood cell analyzer. (B) Serum levels of proinflammatory cytokines and serum iron parameters in 4T1 tumor-bearing mice. Serum was harvested at the time points indicated following injection of 4T1 cells. Serum concentrations of IFN-γ and IL-6 were analyzed by ELISA; serum iron and transferring saturation were measured using an Iron/TIBC reagent set. (C) (D) RBC lifespan of 4T1 tumor-bearing mice. Sulfo-NHS-biotin was injected into mice intravenously on day 7 after tumor cells injection. A drop of blood was collected from the tail on days 8, 15, 22, 29. The percent of biotinylated RBCs was analyzed by flow cytometry. (C) The decay curves are shown for 4 mice in each group. (D) Representative histograms of data from one mouse in each group are shown on day 22 after tumor cells implantation. (E) Serum levels of G-CSF in control and 4T1 tumor-bearing mice. (F) Photograph of femurs dissected from control and 4T1 tumor-bearing mice on day 21 after tumor cells implantation. (G) Total cells of bone marrow (BM) in one femur were counted at indicated times after tumor induction. (H) The percentage of Ter119 and CD71 positive cells of bone marrow in control and 4T1 tumor-bearing mice at indicated times after tumor growth. (I) Representative example of CD71 and Ter119 profiles from the bone marrow of control and 4T1 tumor-bearing mice on day 21 after tumor cells implantation. (J) Graphs show the total number of cell subsets expressing CD71 or Ter119 staining in bone marrow. (K)The numbers of BFU-E and CFU-E derived colonies from the bone marrow of control and 4T1 tumor-bearing mice. All data are expressed as the mean ± SEM; n = 4–6 mice per group for one out of three independent experiments. *p < 0.05, **p < 0.01, ***p < 0.001 compared with control group.

To study the consequences of tumor development on erythropoiesis, initially, we measured RBC numbers, reticulocyte numbers, hemoglobin concentrations and hematocrit in the peripheral blood of 4T1 tumor-bearing mice. Tumor-bearing mice had an anemic phenotype, with lower RBC count, hematocrit and Hb levels, as well as increased reticulocytosis compared to control mice (Fig 1A). We then examined the contribution of inflammation to anemia in our model. Inflammatory reaction is associated with production of proinflammatory cytokines, such as interleukin-6 (IL-6), tumor necrosis factor-α (TNF-α) and interferon-gamma (IFN-γ). IL-6 can induce hepcidin synthesis, which leads to iron-restricted erythropoiesis and anemia [4]. IFN-γ can impair proliferation of hematopoietic stem cells (HSCs) [24] and inhibit erythroid progenitor colony formation [16]. Our results show that the serum levels of IL-6 were only slightly affected by tumor at day 28, while the serum levels of IFN-γ were not increased, and serum TNF-α was undetectable (data not shown) in 4T1 tumor-bearing mice up to day 28 after tumor cells implantation. The serum iron and transferrin saturation also did not change significantly in tumor-bearing mice (Fig 1B). Impaired erythrocyte life cycle is one of the factors contributing to AI [16], thus we tested the possibility that RBC lifespan could be affected in our model. As shown in Fig 1CD, the percent of biotinylated RBCs in tumor-bearing mice is similar to control mice at different time, indicating RBC lifespan is not reduced. Collectively, these findings indicate that the anemia is not due to induction of systemic inflammation in our model. We thus sought to explore the roles of G-CSF in tumor-induced anemia. Serum G-CSF was examined by flow cytometry using the mouse G-CSF Flex-Set bead array. As shown in Fig 1E, serum G-CSF values were significantly increased in tumor-bearing mice in comparison with control mice.

### Medullar erythropoiesis repression is associated with 4T1 tumor development

G-CSF has been shown to block medullar erythropoiesis; therefore we investigated the effect of tumor development on bone marrow erythropoiesis. We observed that the long bones of the tumor-bearing mice were paler than those of control mice (Fig 1F), which was associated with the reduction in the total number of cells in femurs (Fig 1G), suggesting that bone marrow erythropoiesis is affected by tumor growth. Ter119 has been identified as an erythroid lineage-specific marker expressed from the proerythroblast to the mature RBC stage, whereas CD71 expression changes with the degree of maturation. These two markers reflect the erythroblast terminal differentiation [17], and we compared the percentage of Ter119-positive and CD71-postitive cells using flow cytometry. As shown in Fig 1HIJ, the proportions of Ter119+ cells and CD71+ cells in tumor-bearing mice were both significantly lower than in control mice, which indicate tumor-stress impaired the erythroblast terminal differentiation in bone marrow. The RBCs originate from hematopoietic stem cell through several developmental stages, in which the first committed erythroid progenitor is BFU-E that matures into an erythroid colony-forming unit (CFU-E) before extending the proerythroblast stage [1, 18, 23]. Therefore, we measured the numbers of BFU-E and CFU-E by plating bone marrow cells in methylcellulose; both BFU-E and CFU-E colony numbers of bone marrow decreased along with 4T1 tumor growth (Fig 1K), indicating that there are fewer functional erythroid progenitors in bone marrow of tumor-bearing mice. As positive control, treatment of wild-type mice with G-CSF also led to bone marrow erythropoiesis repression (S1 Fig).

### 4T1 tumor induces stress erythropoiesis in spleen

**Fig 2:**
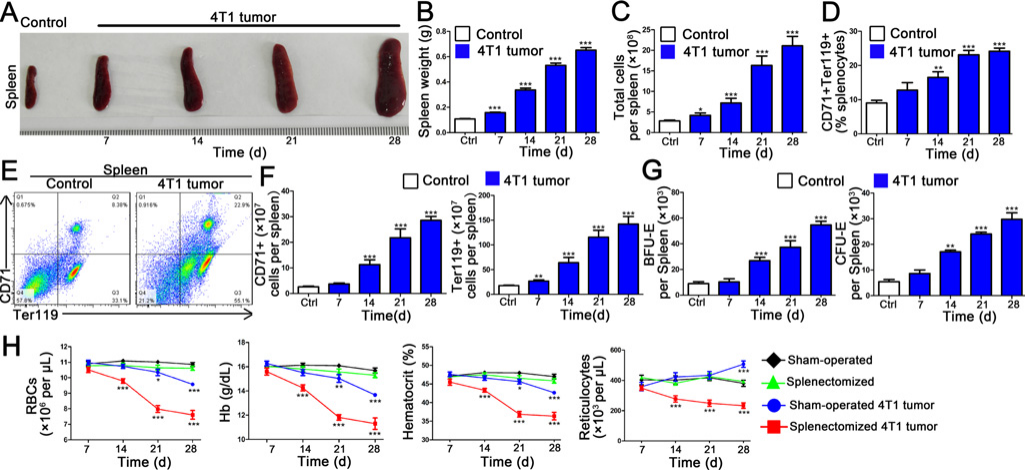
The essential role of spleen in tumor-stress erythropoiesis. (A) (B) (C) Splenomegaly in tumor-bearing mice. (A) Spleens were collected and photographed at the indicated times after tumor cells transplantation. (B) Graph shows the mean spleen weight ± SEM of control and tumor-bearing mice from six to eight mice per time points and is representative of three separate experiments. (C) Splenocytes were counted at indicated times after tumor induction. Each bar represents the mean (±SEM n = 4–6) of triplicate determinations. *p < 0.05, ***p < 0.001, compared with control group. (D) The percentage of Ter119 and CD71 positive cells of spleen in control and 4T1 tumor-bearing mice at indicated times after tumor growth. Each bar represents the mean (±SEM n = 4) of triplicate determinations. **p < 0.01, ***p < 0.001, compared with control group. (E) Representative examples of CD71 and Ter119 profiles from the spleen of control and 4T1 tumor-bearing mice on day 21 after tumor cells transplantation. (F) Numbers of erythroid populations in spleen of control and 4T1 tumor-bearing mice. Each bar represents the mean (±SEM n = 4) of triplicate determinations. **p < 0.01, ***p < 0.001, compared with control group. (G) The numbers of BFU-E and CFU-E derived colonies from the spleen of control and 4T1 tumor bearing mice. Each bar represents the mean (±SEM n = 4) of triplicate determinations. **p < 0.01, ***p < 0.001, compared with control group. (H) BALB/c mice were splenectomized and allowed to recover for at least 2 weeks. Mice with or without spleen were implanted 4T1 cells. Blood was collected at indicated times after tumor cells transplantation. Hematological parameters were analyzed. All data are expressed as the mean ± SEM; n = 6 mice per group for one out of three independent experiments. *p < 0.05, ** p < 0.01, ***p < 0.001, compared with sham-operated control group.

In our experiments, splenomegaly and splenic erythropoiesis developed during G-CSF administration (S2 Fig); therefore we further examined the development of splenomegaly in response to tumor growth by counting cells of spleen and weighing spleens harvested from tumor-bearing mice. One week after 4T1 tumor cells implantation, the average spleen weight and splenocyte numbers increased markedly and continued to increase over subsequent weeks (Fig 2 ABC). We then wondered whether tumors elevate erythropoietic activity of spleen. Thus, we determined the differentiation profiling of terminal erythroid precursors from spleen by flow cytometry. As shown in Fig 2DEF, tumor development triggered increases of CD71+ and Ter119+ cells. Next, we measured the numbers of BFU-E and CFU-E by plating splenocytes in methylcellulose; contrary to bone marrow, the numbers of BFU-E and CFU-E of spleen were increased in the tumor-bearing mice (Fig 2G), indicating that expansion of erythroid progenitors in the spleen responses to tumor burden. Together, these data show that, in tumor stress conditions, the spleen and the bone marrow react in a very different way. Indeed, tumor progression is associated with the suppression of bone marrow erythropoiesis, whereas tumor stress promotes erythropoiesis in the spleen.

### Splenectomy aggravates tumor-induced anemia

To further confirm the crucial role of spleen in stress erythropoiesis of tumor-bearing mice and wild-type mice treated with G-CSF, we analyzed the hematologic parameters in either splenectomized or sham-operated mice. Peripheral blood analysis revealed that splenectomy significantly reduced the number of RBCs, reticulocytes, hematocrit and down regulated the Hb levels in tumor-bearing mice (Fig 2H), indicating that the anemia was of greater severity in the tumor-bearing mice undergoing splenectomy than in the sham-operated tumor-bearing mice. Splenectomy also induced severe anemia in wild-type mice treated with G-CSF (S3 Fig). As control, splenectomized normal mice did not develop an overt peripheral blood anemia, indicating that splenic function is not essential for steady erythropoiesis in normal mice. We also detected the inflammatory cytokines and G-CSF in serum of splenectomized tumor-bearing mice, and we found the results are similar to sham-operated tumor-bearing mice (S4 Fig). These results suggest that G-CSF is involved in tumor-induced anemia, and the spleen is indispensable for tumor-induced stress erythropoiesis.

### Macrophage depletion impairs tumor-driven erythropoiesis in spleen

**Fig 3:**
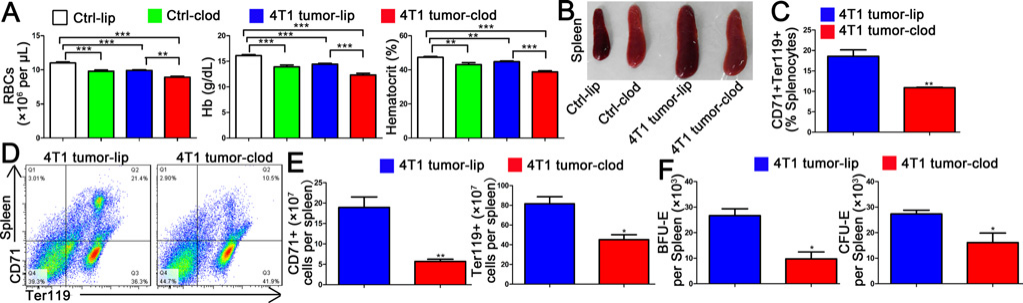
Liposomal clodronate treatment impairs tumor-driven splenic erythropoiesis. (A) Hematological parameters in control mice injection with liposome (ctrl-lip), control mice injection with liposomal clodronate (ctrl-clod), 4T1 tumor-bearing mice injection with liposome (4T1 tumor-lip) and 4T1 tumor-bearing mice injection with liposomal clodronate (4T1 tumor-clod). Tumor cells were implanted into the right fourth mammary gland of female BALB/c mice (day 0). After tumor cells injection, mice were intravenous (i.v.) injected with liposomal clodronate 100 μl on days 7 and 18. On day 28, blood was collected from the mice, and RBC counts, Hb concentrations and hematocrit were measured on the automated blood cell analyzer. All data are expressed as the mean ± SEM; n = 6 mice per group for one out of three independent experiments. **p < 0.01,***p < 0.001. (B) Spleens from ctrl-lip, ctrl-clod, 4T1 tumor-lip and 4T1 tumor-clod mice were collected and photographed on day 20 after tumor cells implantation. (C) The percentage of CD71 and Ter119 positive cells of spleen in 4T1 tumor-lip and 4T1 tumor-clod mice. Each bar represents the mean (±SEM n = 4) of triplicate determinations. **p < 0.01 versus 4T1 tumor-lip. (D) Representative examples of CD71 and Ter119 profiles from the spleen of 4T1 tumor-lip and 4T1 tumor-clod mice. (E) Quantification of the number of CD71 positive or Ter119 positive cells are shown (n = 4 per group). *p < 0.05, **p <0.01 versus 4T1 tumor-lip. (F) The numbers of BFU-E and CFU-E derived colonies from the spleen of 4T1 tumor-lip and 4T1 tumor-clod mice. Each bar represents the mean (±SEM n = 4) of triplicate determinations. *p< 0.05 versus 4T1 tumor-lip.

Liposomal clodronate has been shown to reduce the number of macrophages in spleen and bone marrow [17, 23]. To study the role of macrophages during tumor-stress erythropoiesis, we depleted macrophages in vivo by administering liposomal clodronate on days 7 and 18 of 4T1 tumor cells implantation. By day 28 tumor cells transplantation, clodronate treatment decreased RBC numbers, hemoglobin levels and hematocrit, consistent with the weak color of spleen (Fig 3AB). We also found that absence of macrophages impacted splenic erythroid terminal differentiation using flow cytometry with CD71 and Ter119 markers at day 20 of tumor cells transplantation (Fig 3CDE). On day 20 tumor cells transplantation, splenocytes were isolated and the BFU-E and CFU-E were analyzied. Macrophage depletion reduced the numbers of splenic-stress BFU-E and CFU-E (Fig 3F), indicating that there are fewer functional erythroid progenitors in the spleen under the conditions of macrophage deficiency. These results demonstrate that macrophages functionally contribute to splenic erythropoiesis in tumor-bearing mice.

### Tumor-stress increases the production of Epo

**Fig 4:**
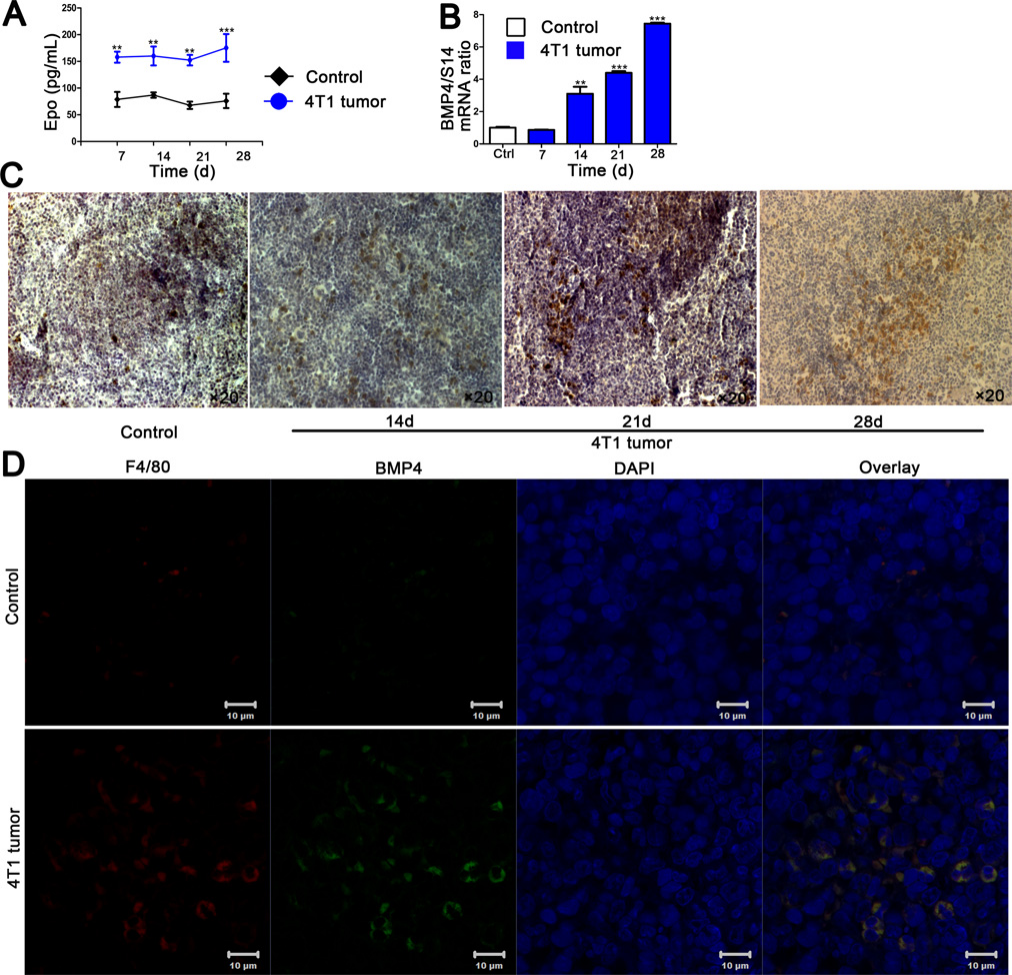
Tumor growth alters serum Epo levels and splenic microenvironment of mice. (A)Serum levels of Epo in control and 4T1 tumor-bearing mice at indicated times after tumor induction. (B) Quantification of spleen BMP4 mRNA by quantitative reverse-transcribed polymerase chain reaction, normalized to S14 mRNA. (C) Analysis of spleen BMP4 by immunochemistry. Spleens were collected at the indicated times after 4T1 tumor cells implantation. Representative images of BMP4 staining from spleens of control and 4T1 tumor-bearing mice were photographed by microscope. (D) Confocal analysis of F4/80 and BMP4 colocalization in spleen macrophages. Spleens were collected on day 21 after tumor cells transplantation. Representative images of F4/80, BMP4, and DAPI staining sections from spleens of control and 4T1 tumor-bearing mice were photographed by confocal microscope. Data are means ± SEM (n = 4–6) calculated from three independent experiments. **p<0.01, ***p<0.001 compared with control group.

Epo, a hormone that stimulates erythropoiesis, has been reported to mediate induction of BMP4 by spleen macrophages [16]. And BMP4 promotes the erythroid progenitors differentiation into BFU-E, which works in concern with Epo [15]. To further study the mechanisms underlying macrophages mediated-erythropoiesis in the spleen of tumor-bearing mice, we measured circulating Epo levels. Epo concentrations elevated in the serum of mice with tumor burden compared to normal controls, suggesting that tumor-stress stimulates Epo expression (Fig 4A).

### BMP4 expression is associated with splenic macrophages in tumor-bearing mice

**Fig 5:**
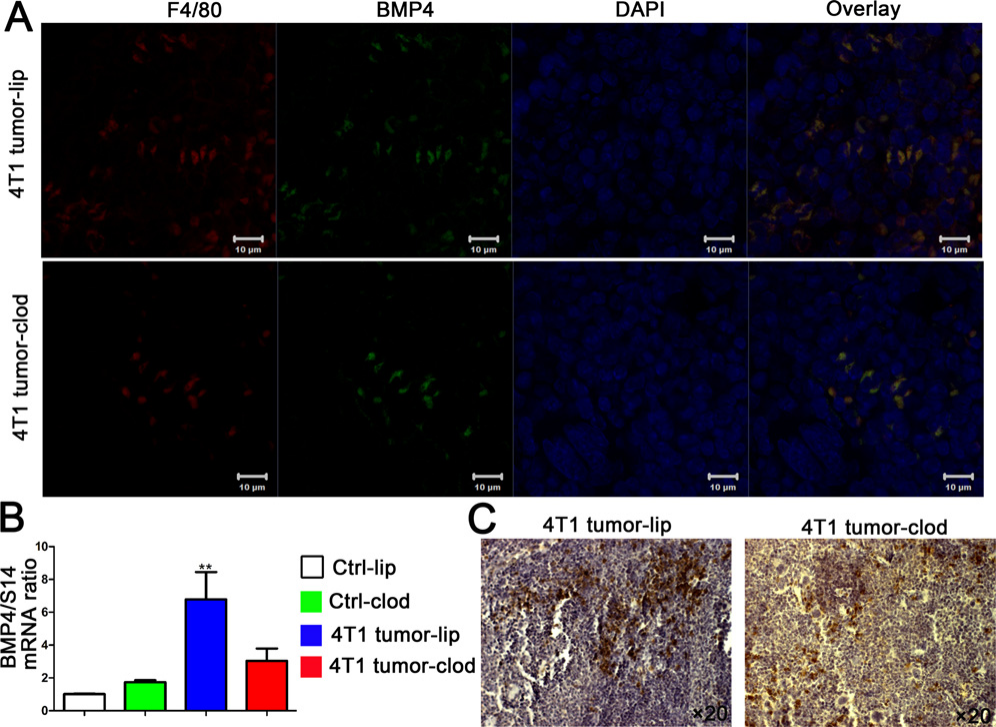
Macrophage depletion with clodronate decreases the expression of splenic BMP4 in tumor-bearing mice. After tumor cells injection, mice were intravenous (i.v.) injected with liposomal clodronate. Spleens were harvested on day 20 after tumor cells implantation. (A) Confocal analysis of F4/80 and BMP4 colocalization in spleen macrophages. Representative images of F4/80, BMP4, and DAPI staining from spleens of 4T1 tumor-lip and 4T1 tumor-clod mice were photographed by confocal microscope. (B) Quantification of spleen BMP4 mRNA in control mice injection with liposome (ctrl-lip), control mice injection with liposomal clodronate (ctrl-clod), 4T1 tumor-bearing mice injection with liposome (4T1 tumor-lip) and 4T1 tumor-bearing mice injection with liposomal clodronate (4T1 tumor-clod) by quantitative reverse-transcribed polymerase chain reaction, normalized to S14 mRNA. Each bar represents the mean ± SEM (n = 3) of three independent experiments. **p < 0.01, compared with Ctrl-lip group. (C) Analysis of spleen BMP4 by immunochemistry on day 20 after tumor cells transplantation. Representative images of BMP4 staining from spleens of 4T1 tumor-lip and 4T1 tumor-clod mice were photographed by microscope.

Since BMP4 has been shown to promote the development of stress erythroid progenitors in spleen [15, 18] and we witnessed an erythropoietic response in the spleen of tumor-bearing mice, we assessed BMP4 expression in the spleen of 4T1 breast tumor model; we found that BMP4 in the spleen was increased at the mRNA and protein level under tumor-stress conditions (Fig 4BC). Splenic macrophages have been previously suggested as the source of BMP4 [16, 23]. In an attempt to explore the contributions of macrophages for BMP4 produce in tumor-stress conditions, we analyzed colocalization of BMP4 and F4/80 by confocal immunofluorescence. Double staining for F4/80 and BMP4 showed that the majority of spleen macrophages expressing F4/80 were associated with BMP4 expression in tumor-bearing mice (Fig 4D). Furthermore, macrophage depletion with cloldronate reduced the induction of splenic BMP4 (Fig 5), consist with the anemia and the decreased numbers of splenic-stress BFU-E and CFU-E in 4T1 tumor-bearing mice treated with clodronate. These data indicate that macrophages from the spleen synthesize BMP4 and then stimulate the formation of BFU-E in response to tumor-induced anemia.

### Splenectomy and liposomal clodronate inhibit tumor growth

**Fig 6:**
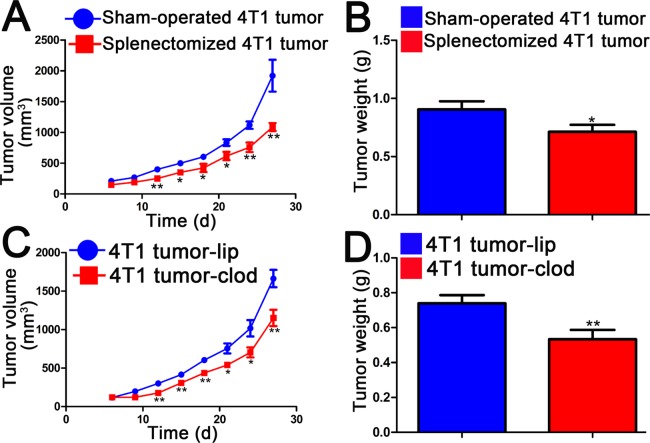
Splenectomy and liposomal clodronate treatment inhibit growth of 4T1 mammary tumors. (A)(B) BALB/c mice were splenectomized and allowed to recover for at least 2 weeks. 4T1 tumor cells were injected into the right fourth mammary gland of mice with or without spleen. (A)Graph depicting tumor volume from the sixth day of tumor cells injection in each group. (B) Tumor weight was determined on day 28 after tumor cells implantation. Each bar represents the mean ± SEM. n = 8–10 mice per group for one out of three independent experiments. *p < 0.05, **p < 0.01, compared with sham-operated tumor-bearing mice. (C) (D) 4T1 tumor-bearing mice were injected with liposome (4T1 tumor-lip) and liposomal clodronate (4T1 tumor-clod). (C)Tumor volume was recorded for 28 days from day 6 after tumor cells implantation, data are plotted with mean tumor volume in mm^3^. (D) After 28 days, the tumors were excised from mice and weighted. Data represent the mean ± SEM (n = 6) of three independent experiments. *p < 0.05, **p < 0.01, compared with 4T1 tumor-lip group.

The major function of red blood cells is to transport hemoglobin, which carries oxygen from the lungs to tissues. Rapid tumor growth often results in hypoxia, which in turn impacts the tumor progression [19, 25]. In our experiments, splenectomy and clodronate treatment abolish splenic erythropoiesis and aggravate 4T1 tumor-induced anemia. We next asked whether tumor growth was affected by splenic erythropoiesis. As shown in Fig 6, splenectomy or pharmacological inhibition of splenic erythropoiesis attenuates 4T1 tumor growth. These findings suggest that pathological splenic erythropoiesis promotes the growth of 4T1 breast tumors.

## Discussion

Anemia is a debilitating condition that can be complicated by diverse causes. As a primary consequence of tumor burden, cancer-related anemia (CRA) has been described in cancer patients and animal cancer models. The pathogenesis of the CRA in patients not receiving cancer therapy is complex; many factors have been implicated, such as functional iron deficiency, erythropoietin deficiency, impaired bone marrow erythropoiesis and shortened red blood cell lifespan. There is considerable interest in therapies for CRA; iron therapy, erythropoietic-stimulating agents, and RBC transfusions are the most common treatment options for CRA [6, 7]. However, these therapies are restricted and controversial; therefore there is growing interest in characterizing new factors of CRA and finding new targets for CRA treatment. Here, using mouse breast tumor model, we show that 4T1 tumor-bearing mice display G-CSF-mediated anemia; importantly, we found that splenectomy accelerates and aggravates tumor-induced anemia. As the major regulator of in vivo granulopoiesis, G-CSF is widely administered to patients who recover from chemotherapy to increase the immature granular cells and to reduce the severity and duration of neutropenia [26, 27]. G-CSF also has been routinely used to therapeutically mobilize hematopoietic stem cells (HSCs) to the bloodstream for transplantation [28]. HSCs reside in specialized bone marrow niches that are maintained by bone marrow macrophages. G-CSF has been shown to disrupt bone marrow macrophages and HSC niches, which mobilizes HSC to the blood [29, 30] and blocks medullar erythropoiesis [14]. HSCs produce all cells that replenish the blood and immune systems, and HSCs mobilization may induce anemia; indeed Papaldo and colleagues found that G-CSF administration worsen anemia in early breast cancer patients treated with chemotherapy agents [31]. However, strong evidences are needed to further clarify weather G-CSF is involved in tumor-induced anemia. Given myeloid cells can secrete high levels of G-CSF [32], future studies need to confirm serum G-CSF of tumor-bearing mice was mainly synthesized by tumor cells or myeloid cells.

Spleen, a unique lymphoid organ, combines the innate and adaptive immune system. In addition, spleen plays key roles in maintaining the balance of erythropoiesis, such as removing older erythrocytes from circulation and recycling iron [33]. Spleen can adjust its microenvironment to harbor HSCs [32] and support stress erythropoiesis in anemia state [15–18]. In the present work, we show that erythropoiesis in spleen and bone marrow exhibited a different behavior in mice with tumor burden; medullar erythropoiesis repression was associated with tumor progression, conversely, splenic erythropoiesis was stimulated under tumor-stress conditions. Considering that HSCs can be mobilized by G-CSF from the bone marrow and accumulate in the spleen [32], it will be interesting to investigate the crosstalk between spleen and bone marrow in mice with tumor development.

Macrophages, which are classified as immunological cells, play various roles in innate and adaptive immune system [34]. Several lines of evidence suggest that macrophages regulate the erythroid development in bone marrow and spleen [16, 17, 20–23]. The contribution of macrophages to pathological erythropoiesis in polycythemia vera and β-thalassemia has been suggested by Revella and colleagues. [17] The in vivo relevance of splenic microenvironment for developing RBCs in tumor-bearing mice has been unclear, and the function of macrophages to pathological erythropoiesis of tumor-bearing mice has not been investigated. In this study, we reported a decisive contribution of spleen resident macrophages to splenic erythropoiesis in mice with tumor development, indicating that to promote stress erythropoiesis, pathological changes may happen in splenic macrophages under tumor development. The solid tumors require an ample blood supply for expansive growth. Macrophages mediated erythropoiesis in spleen may accelerates tumor progression by improving the oxygen and nutrient supply; indeed tumor-associated macrophages (TAMs) have been observed in a variety of human tumors [34–37], and spleen-derived TAMs contribute to tumor growth [38]. In this study, splenectomy or depletion of macrophages with clodronate suppresses the tumor growth. Our findings suggest that macrophages promote tumor growth at least partly through simulating splenic erythropoiesis that leads to increased oxygen perfusion and improved tumor-associated anemia. BMP4, a member of the transforming growth factor beta (TGF-β) superfamily of growth factors, has been suggested to regulate HSCs development [39] and promote stress erythropoiesis [40]. Of interest, our results indicate that splenic macrophages synthesize bone morphogenetic protein 4(BMP4) in response to tumor stress. Liposomal clodronate treatment impairs BMP4 induction, delays development of stress BFU-E of spleen and severely reduces peripheral erythroid in tumor-bearing mice, consistent with the requirement of macrophages to mount BMP4-mediated stress erythropoiesis. Our results also suggest the importance of Epo in the promotion of tumor-driven erythropoiesis in the spleen, as the serum Epo kept high levels in tumor-bearing mice.

In conclusion, to our knowledge, this is the first evaluating impact of macrophages on splenic erythropoiesis induced by G-CSF in murine tumor model. Our study contributes important insights in tumor-induced anemia and tumor-stress erythropoiesis, and indicates that macrophages, BMP4 and Epo are important factors for splenic erythropoiesis in tumor-bearing mice. Further research in this topic will open up new fields for understanding tumor-induced anemia and for identifying potential therapy strategies for cancer.

## Supporting Information

S1 Fig G-CSF impaires the erythropoiesis in bone marrow. BALB/c mice were treated with outside source G-CSF for 8 d (250 μg/kg/d). (A) Femurs from control and G-CSF-treated mice. (B) Total cell numbers of bone marrow (BM) in control and G-CSF-treated mice. (C) Representative examples of CD71 and Ter119 profiles from the bone marrow (BM) of control and G-CSF-treated mice. (D) The percentage of CD71 and Ter119 positive cells of bone marrow (BM) in control and G-CSF treated mice. (E) Numbers of erythroid populations in bone marrow of control and G-CSF treated mice. (F) The numbers of BFU-E and CFU-E derived colonies from the bone marrow of control and G-CSF treated mice. Each bar represents the mean (±SEM n = 4) of triplicate determinations. *p < 0.05, **p < 0.01, ***p < 0.001, compared with control group.
(TIF)

S2 Fig G-CSF induces splenic erythropoiesis. BALB/c mice were treated with outside source G-CSF for 8 d (250 μg/kg/d). (A) Spleens from control and G-CSF-treated mice. (B) Spleen weight and total cell numbers of spleen in control and G-CSF-treated mice. (C) Representative examples of CD71 and Ter119 profiles from the spleen of control and G-CSF-treated mice. (D) The percentage of CD71 and Ter119 positive cells of spleen in control and G-CSF treated mice. (E) The total numbers of CD71 or Ter119 positive cell in the spleen from control and G-CSF-treated mice. (F) The numbers of BFU-E and CFU-E derived colonies from the spleen of control and G-CSF treated mice. Each bar represents the mean (±SEM n = 4) of triplicate determinations. *p < 0.05, **p < 0.01, ***p < 0.001, compared with control group.
(TIF)

S3 Fig Splenectomy aggravates G-CSF-induced anemia. Hematological parameters in G-CSF-treated mice with or without spleen (250μg/kg/d G-CSF for 21d). Blood was collected from the mice, and RBC counts, reticulocytes, hemoglobin (Hb) concentrations and hematocrit were measured on the automated blood cell analyzer. All data are expressed as the mean ± SEM; n = 6 mice per group for one out of three independent experiments. *p < 0.05, ***p < 0.001.
(TIF)

S4 Fig Serum levels of proinflammatory cytokines, serum iron parameters and serum levels of G-CSF in splenectomized 4T1 tumor-bearing mice. BALB/c mice were splenectomized and allowed to recover for at least 2 weeks. Mice were implanted 4T1 cells and serum was harvested at day 28 after tumor cells transplantation. (A) Serum concentrations of IFN-γ and IL-6 were analyzed by ELISA; serum iron and transferring saturation were measured using an Iron/TIBC reagent set. Each bar represents the mean (±SEM n = 4) of triplicate determinations. (B) Serum levels of G-CSF in splenectomized 4T1 tumor-bearing mice were analyzed by flow cytometry using the mouse G-CSF Flex-Set bead array at indicated times. Data represents the mean (±SEM n = 4) of triplicate determinations.
(TIF)
