# Supplementary figures and images for: Macrophages Support Splenic Erythropoiesis in 4T1 Tumor-Bearing Mice
Source: PLoS One. 2015 Mar 30;10(3):e0121921. doi: 10.1371/journal.pone.0121921 (PMC4378955; doi:10.1371/journal.pone.0121921)

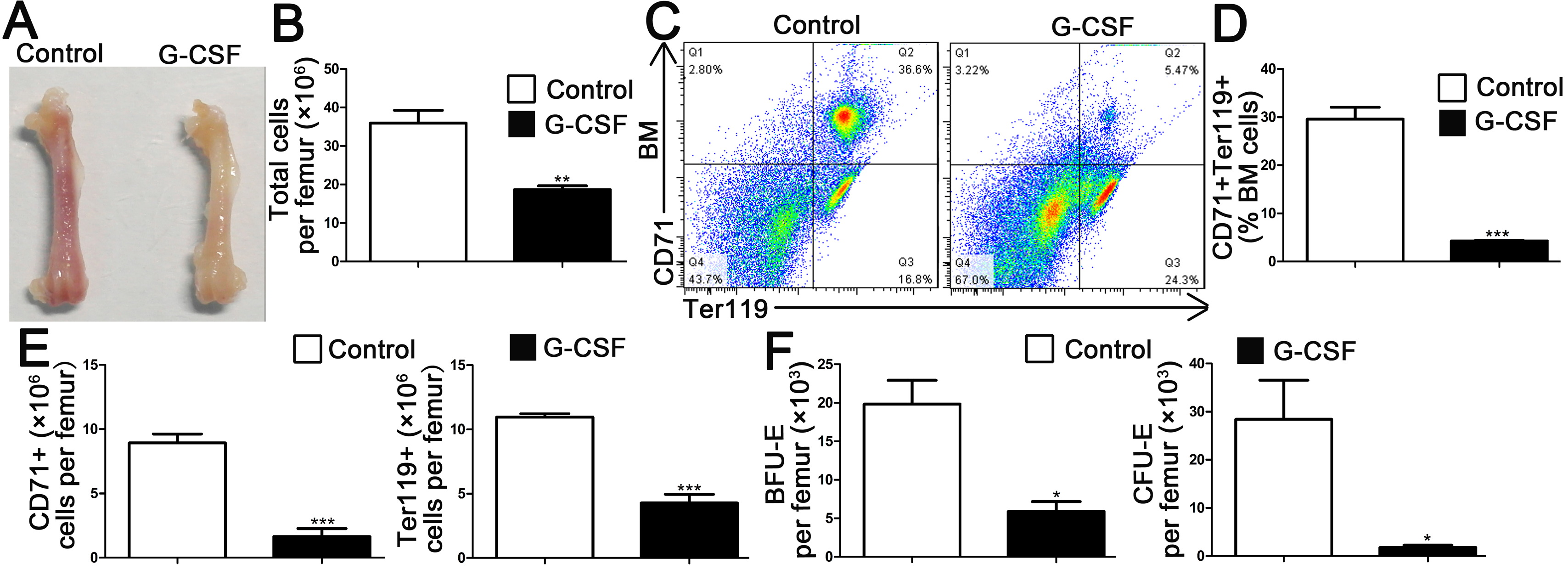

Supplement: S1 Fig — BALB/c mice were treated with outside source G-CSF for 8 d (250 μg/kg/d). (A) Femurs from control and G-CSF-treated mice. (B) Total cell numbers of bone marrow (BM) in control and G-CSF-treated mice. (C) Representative examples of CD71 and Ter119 profiles from the bone marrow (BM) of control and G-CSF-treated mice. (D) The percentage of CD71 and Ter119 positive cells of bone marrow (BM) in control and G-CSF treated mice. (E) Numbers of erythroid populations in bone marrow of control and G-CSF treated mice. (F) The numbers of BFU-E and CFU-E derived colonies from the bone marrow of control and G-CSF treated mice. Each bar represents the mean (±SEM n = 4) of triplicate determinations. *p < 0.05, **p < 0.01, ***p < 0.001, compared with control group. (TIF) [file pone.0121921.s001.tif]

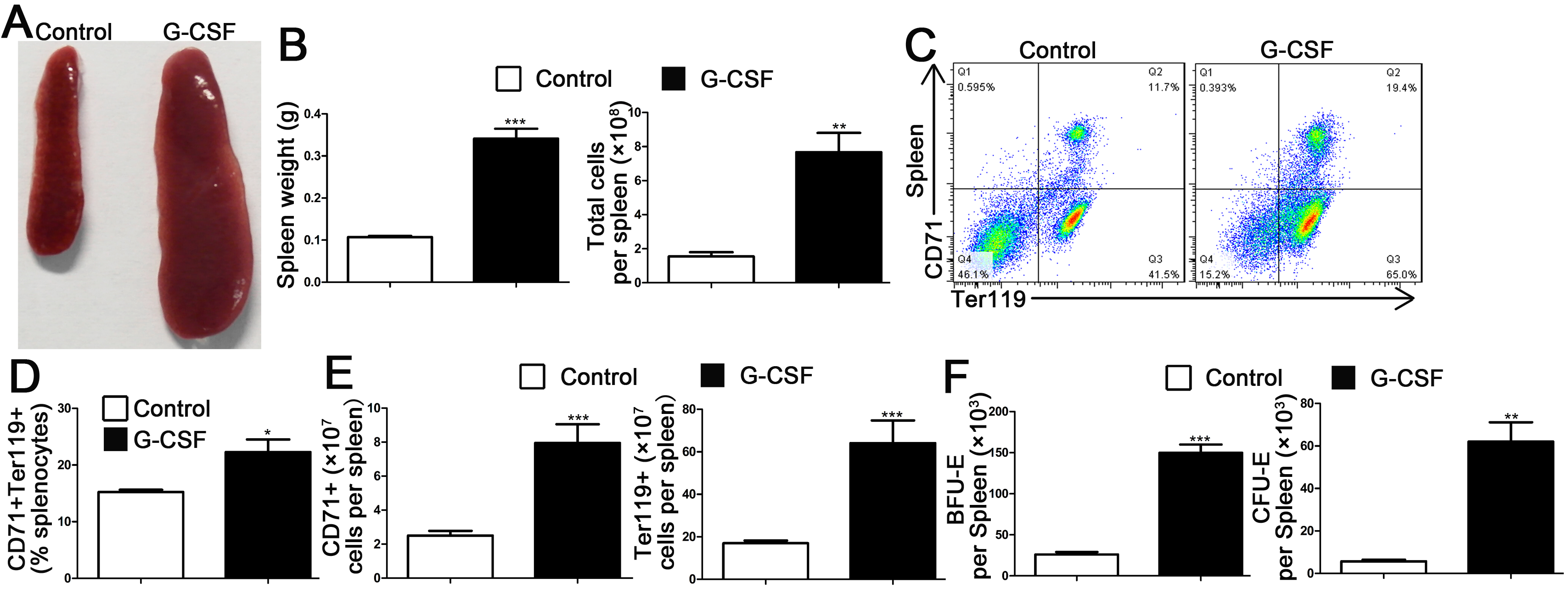

Supplement: S2 Fig — BALB/c mice were treated with outside source G-CSF for 8 d (250 μg/kg/d). (A) Spleens from control and G-CSF-treated mice. (B) Spleen weight and total cell numbers of spleen in control and G-CSF-treated mice. (C) Representative examples of CD71 and Ter119 profiles from the spleen of control and G-CSF-treated mice. (D) The percentage of CD71 and Ter119 positive cells of spleen in control and G-CSF treated mice. (E) The total numbers of CD71 or Ter119 positive cell in the spleen from control and G-CSF-treated mice. (F) The numbers of BFU-E and CFU-E derived colonies from the spleen of control and G-CSF treated mice. Each bar represents the mean (±SEM n = 4) of triplicate determinations. *p < 0.05, **p < 0.01, ***p < 0.001, compared with control group. (TIF) [file pone.0121921.s002.tif]

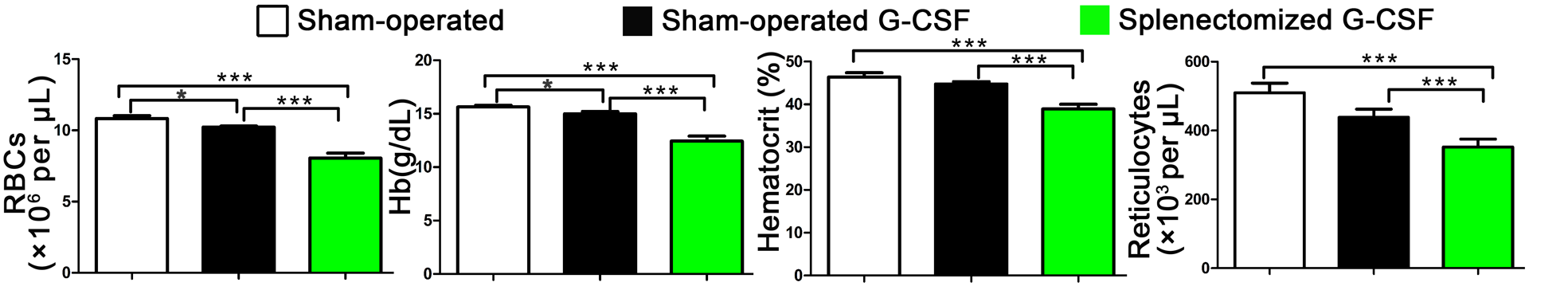

Supplement: S3 Fig — Hematological parameters in G-CSF-treated mice with or without spleen (250μg/kg/d G-CSF for 21d). Blood was collected from the mice, and RBC counts, reticulocytes, hemoglobin (Hb) concentrations and hematocrit were measured on the automated blood cell analyzer. All data are expressed as the mean ± SEM; n = 6 mice per group for one out of three independent experiments. *p < 0.05, ***p < 0.001. (TIF) [file pone.0121921.s003.tif]

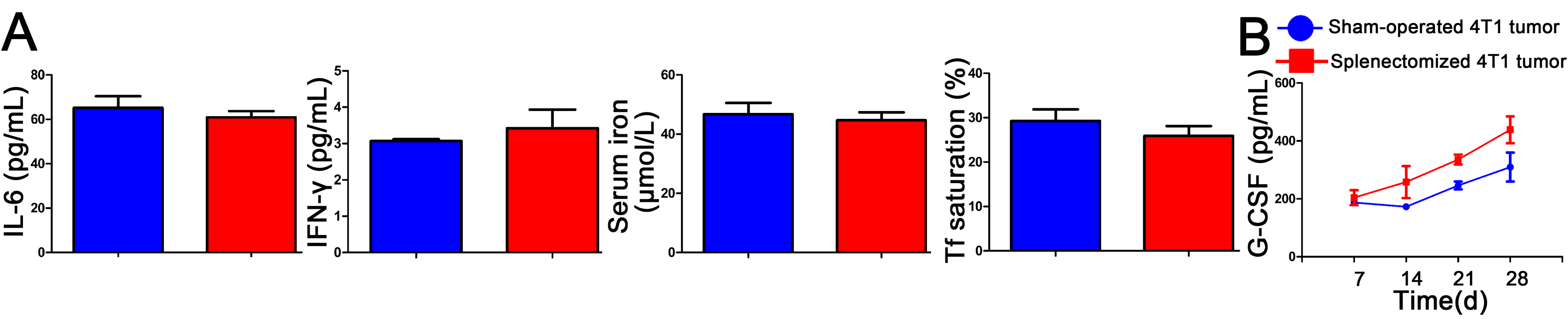

Supplement: S4 Fig — BALB/c mice were splenectomized and allowed to recover for at least 2 weeks. Mice were implanted 4T1 cells and serum was harvested at day 28 after tumor cells transplantation. (A) Serum concentrations of IFN-γ and IL-6 were analyzed by ELISA; serum iron and transferring saturation were measured using an Iron/TIBC reagent set. Each bar represents the mean (±SEM n = 4) of triplicate determinations. (B) Serum levels of G-CSF in splenectomized 4T1 tumor-bearing mice were analyzed by flow cytometry using the mouse G-CSF Flex-Set bead array at indicated times. Data represents the mean (±SEM n = 4) of triplicate determinations. (TIF) [file pone.0121921.s004.tif]
